# The genome sequence of the European Free-tailed Bat, *Tadarida teniotis* (Rafinesque, 1814) (Chiroptera: Molossidae)

**DOI:** 10.12688/wellcomeopenres.27076.1

**Published:** 2026-07-21

**Authors:** Manuel Ruedi, Sonja C. Vernes, Emma C. Teeling, Meike Mai, Bat1K Genome Consortium, Wellcome Sanger Institute Tree of Life Management, Samples and Laboratory team, Wellcome Sanger Institute Scientific Operations: Sequencing Operations, Wellcome Sanger Institute Tree of Life Core Informatics team, Tree of Life Core Informatics collective, Darwin Tree of Life Consortium

**Affiliations:** 1 Muséum d’histoire naturelle de Genève, Geneva, Switzerland; 2 The University of St Andrews, St Andrews, Scotland, UK; 3 School of Biology and Environmental Science, University College Dublin, Dublin, Ireland; 4 Tree of Life Programme, Wellcome Sanger Institute, Hinxton, England, UK

**Keywords:** Tadarida teniotis, European Free-tailed Bat, genome sequence, chromosomal, Chiroptera

## Abstract

We present a genome assembly from an individual female *Tadarida teniotis* (European Free-tailed Bat; Chordata; Mammalia; Chiroptera; Molossidae). The assembly contains two haplotypes with total lengths of 2 587.88 megabases and 2 416.25 megabases. Most of haplotype 1 (94.1%) and haplotype 2 (97.35%) are scaffolded into 24 chromosomal pseudomolecules, including the X sex chromosome. The mitochondrial genome has also been assembled, with a length of 16.88 kilobases. This assembly was generated as part of the Darwin Tree of Life project, which produces genomes for eukaryotic species found in Britain and Ireland, and the Bat1K Project, which aims to sequence the genomes of all living bat species.

## Species taxonomy

Eukaryota; Opisthokonta; Metazoa; Eumetazoa; Bilateria; Deuterostomia; Chordata; Craniata; Vertebrata; Gnathostomata; Teleostomi; Euteleostomi; Sarcopterygii; Dipnotetrapodomorpha; Tetrapoda; Amniota; Mammalia; Theria; Eutheria; Boreoeutheria; Laurasiatheria; Chiroptera; Yangochiroptera; Molossidae; *Tadarida*; *Tadarida teniotis* (Rafinesque, 1814) (NCBI:txid59483).

## Background

The European Free-tailed Bat ( *Tadarida teniotis*) is the sole representative of the family Molossidae occurring in our region. As indicated by its common name, this species is distinguished by a long tail that extends largely beyond the interfemoral membrane. With a wingspan of approximately 45 cm and an adult body mass ranging from 30 to 50 g, it is among the largest bat species in Europe. It is characterised by broad ears and a muzzle with distinctly wrinkled lips, giving it a dog-like facial appearance. The pelage is short and soft, greyish brown dorsally and slightly paler ventrally.

This species is a specialised predator of moths, using low-frequency echolocation calls that are audible to the human ear ( Rydell & Arlettaz, 1994). It is the only European bat species known not to undergo torpor and remains active throughout the winter, a trait that restricts its distribution to thermally favourable regions ( Arlettaz *et al.*, 2000). Its range extends across the Mediterranean Basin eastwards to the Caucasus and the Caspian Sea. During the daytime, the European Free-tailed Bat occupies roosts in narrow crevices, including those in warm cliff faces and rocky gorges, as well as cavities in tall buildings and beneath stone bridges.

The European Free-tailed Bat is listed by the IUCN Red List of Threatened Species as being of Least Concern.

The genome described here is derived from an adult female *Tadarida teniotis* found in the city of Geneva, Switzerland, on 26 October 2022, with a crushed wing that necessitated euthanasia.

## Methods

### Sample acquisition

The specimen used for genome sequencing was an adult female *Tadarida teniotis* (specimen ID SAN27000001, ToLID mTadTen1; Figure 1), collected from Geneva, Switzerland (latitude 46.195, longitude 6.1436) on 2022-10-26. The specimen was found with a crushed wing and was euthanased. It was collected and identified by Manuel Ruedi (Natural History Museum of Geneva). Following dissection, the carcass was deposited in the collections of the Natural History Museum of Geneva under voucher number MHNG-MAMO-3010.048. Organs were immediately frozen on dry ice and stored at –80 °C. Species identification was confirmed by Manuel Ruedi based on morphological characteristics and mitochondrial DNA sequences (GenBank accessions OQ885422 and OQ706681) ( Ruedi *et al.*, 2023). The mTadTen1 sample was used for PacBio HiFi, Hi-C and RNA sequencing.

**Figure 1.**
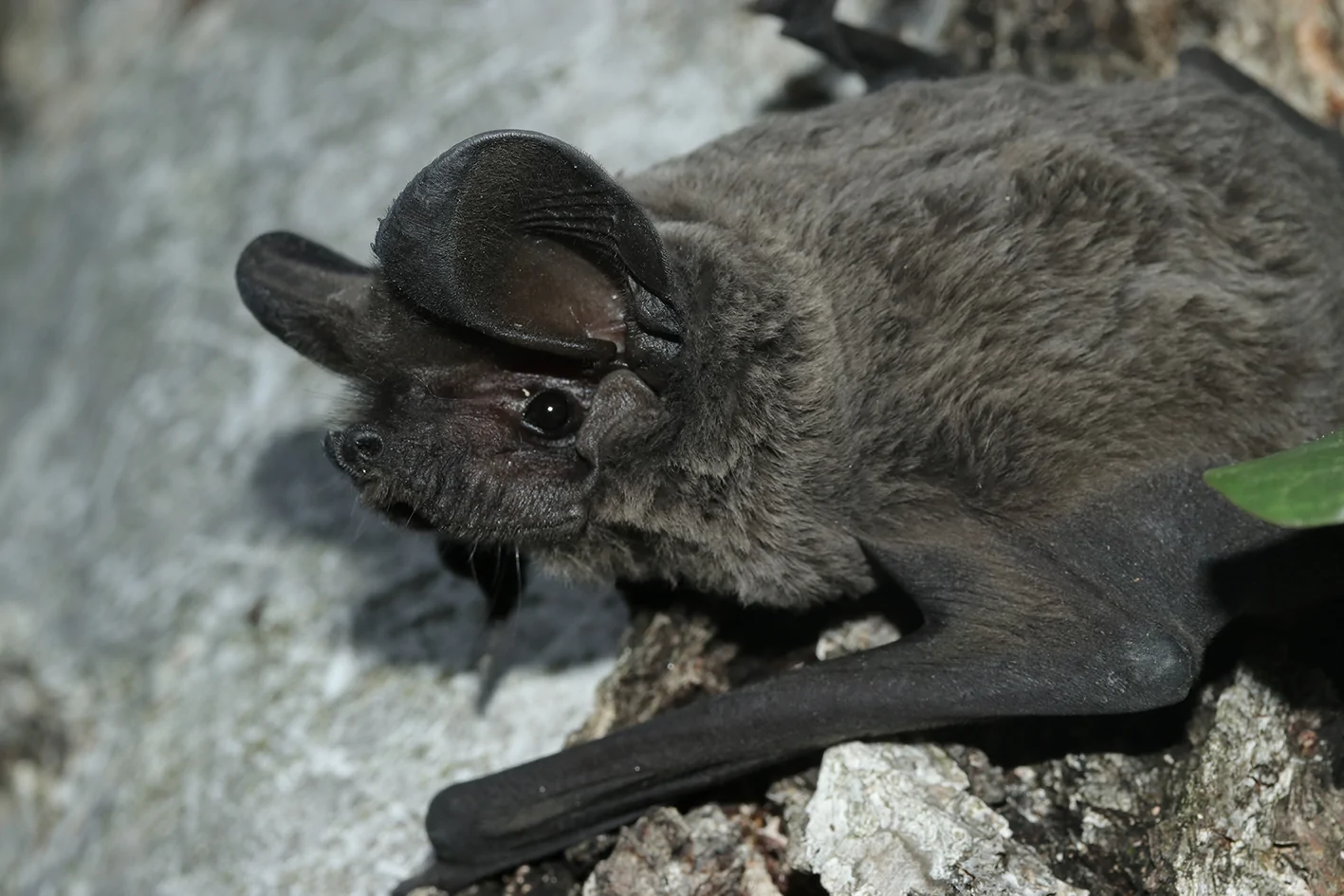
Photograph of the *Tadarida teniotis* (mTadTen1) specimen used for genome sequencing.

### Nucleic acid extraction

Detailed protocols for nucleic acid extraction developed at the Wellcome Sanger Institute (WSI) Tree of Life Core Laboratory are available on protocols.io ( Howard *et al.*, 2025). The mTadTen1 specimen was weighed and triaged to determine the appropriate extraction protocol.

For high molecular weight (HMW) DNA extraction, 24.0 mg of heart tissue was used. The tissue was homogenised by powermashing using a PowerMasher II tissue disruptor. HMW DNA was extracted using the Sanger Tree of Life HMW DNA Extraction: Manual Tissue Nanobind v.2 protocol. DNA was sheared into an average fragment size of 12–20 kb following the Megaruptor®3 for LI PacBio protocol. Sheared DNA was purified by manual SPRI (solid-phase reversible immobilisation). The concentration of the sheared and purified DNA was assessed using a Nanodrop spectrophotometer and Qubit Fluorometer using the Qubit dsDNA High Sensitivity Assay kit. Fragment size distribution was evaluated by running the sample on the FemtoPulse system. For this sample, the final post-shearing DNA had a Qubit concentration of 23.6 ng/μL, with a fragment size of 20.6 kb. The 260/280 spectrophotometric ratio was 1.89, and the 260/230 ratio was 2.14.

RNA was extracted from spleen tissue of the mTadTen1 specimen in the Tree of Life Laboratory at the WSI using the RNA Extraction: Automated MagMax™ *mir*Vana protocol. The RNA concentration was assessed using a Nanodrop spectrophotometer and a Qubit Fluorometer using the Qubit RNA Broad-Range Assay kit. Analysis of the integrity of the RNA was done using the Agilent RNA 6000 Pico Kit and Eukaryotic Total RNA assay.

### PacBio HiFi library preparation and sequencing

Library preparation and sequencing were performed at the WSI Scientific Operations core. Libraries were prepared using the SMRTbell Prep Kit 3.0 (Pacific Biosciences, California, USA), following the manufacturer’s instructions. The kit includes reagents for end repair/A-tailing, adapter ligation, post-ligation SMRTbell bead clean-up, and nuclease treatment. Size selection and clean-up were performed using diluted AMPure PB beads (Pacific Biosciences). DNA concentration was quantified using a Qubit Fluorometer v4.0 (ThermoFisher Scientific) and the Qubit 1X dsDNA HS assay kit. Final library fragment size was assessed with the Agilent Femto Pulse Automated Pulsed Field CE Instrument (Agilent Technologies) using the gDNA 55 kb BAC analysis kit.

The sample was sequenced on a Revio instrument (Pacific Biosciences, California, USA). Prepared libraries with a molarity greater than 2 nM were normalised to 2 nM, and 15 μL was used for making complexes. For libraries with a molarity below 2 nM, the available 10 μL library volume was used without normalisation. Primers were annealed and polymerases were bound to generate circularised complexes, following the manufacturer’s instructions. Complexes were purified using 1.2× SMRTbell beads, diluted to the Revio loading concentration of 200–300 pM, and spiked with a Revio sequencing internal control. The sample was sequenced on a Revio 25M SMRT cell. SMRT Link software (Pacific Biosciences), a web-based workflow manager, was used to configure and monitor the run and to carry out primary and secondary data analysis.

### Hi-C

***Sample preparation and crosslinking***

The Hi-C sample was prepared from 20–50 mg of frozen muscle tissue from the mTadTen1 sample using the Arima-HiC v2 kit (Arima Genomics). Following the manufacturer’s instructions, tissue was fixed and DNA crosslinked using TC buffer to a final formaldehyde concentration of 2%. The tissue was homogenised using the Diagnocine Power Masher-II. Crosslinked DNA was digested with a restriction enzyme master mix, biotinylated, and ligated. Clean-up was performed with SPRISelect beads before library preparation. DNA concentration was measured with the Qubit Fluorometer (Thermo Fisher Scientific) and Qubit HS Assay Kit. The biotinylation percentage was estimated using the Arima-HiC v2 QC beads.

***Hi-C library preparation and sequencing***

Biotinylated DNA constructs were fragmented using a Covaris E220 sonicator and size selected to 400–600 bp using SPRISelect beads. DNA was enriched with Arima-HiC v2 kit Enrichment beads. End repair, A-tailing, and adapter ligation were carried out with the NEBNext Ultra II DNA Library Prep Kit (New England Biolabs), following a modified protocol where library preparation occurs while DNA remains bound to the Enrichment beads. Library amplification was performed using KAPA HiFi HotStart mix and a custom Unique Dual Index (UDI) barcode set (Integrated DNA Technologies). Depending on sample concentration and biotinylation percentage determined at the crosslinking stage, libraries were amplified with 10–16 PCR cycles. Post-PCR clean-up was performed with SPRISelect beads. Libraries were quantified using the AccuClear Ultra High Sensitivity dsDNA Standards Assay Kit (Biotium) and a FLUOstar Omega plate reader (BMG Labtech).

Prior to sequencing, libraries were normalised to 10 ng/μL. Normalised libraries were quantified again to create equimolar and/or weighted 2.8 nM pools. Pool concentrations were checked using the Agilent 4200 TapeStation (Agilent) with High Sensitivity D500 reagents before sequencing. Sequencing was performed using paired-end 150 bp reads on the Illumina NovaSeq X.

### RNA library preparation and sequencing

Libraries were prepared using the NEBNext® Ultra II Directional RNA Library Prep Kit for Illumina (New England Biolabs), following the manufacturer’s instructions. Poly(A) mRNA was isolated from 100 ng of total RNA using oligo (dT) beads, converted to cDNA, and uniquely indexed; 14 PCR cycles were performed. Libraries were prepared with an intended fragment size of 100–300 bp. Libraries were quantified, normalised, and pooled equimolarly to a final concentration of 2.8 nM before dilution to 150 pM for loading. Sequencing was carried out on the Illumina NovaSeq X, generating paired-end reads.

### Genome assembly

Prior to assembly of the PacBio HiFi reads, a database of *k*-mer counts ( *k* = 31) was generated from the filtered reads using FastK. GenomeScope2 ( Ranallo-Benavidez *et al.*, 2020) was used to analyse the *k*-mer frequency distributions, providing estimates of genome size, heterozygosity, and repeat content.

The HiFi reads were assembled using Hifiasm in Hi-C phasing mode ( Cheng *et al.*, 2021, 2022), producing two haplotypes. Hi-C reads ( Rao *et al.*, 2014) were mapped to the primary contigs using bwa-mem2 ( Vasimuddin *et al.*, 2019). Contigs were further scaffolded with Hi-C data in YaHS ( Zhou *et al.*, 2023), using the --break option for handling potential misassemblies. The scaffolded assemblies were evaluated using Gfastats ( Formenti *et al.*, 2022), BUSCO ( Manni *et al.*, 2021) and MerquryFK ( Rhie *et al.*, 2020).

The mitochondrial genome was assembled using MitoHiFi ( Uliano-Silva *et al.*, 2023). The MitoHiFi reference was the mitochondrial genome of *Tadarida teniotis* (NC_036330.1).

### Assembly curation

The assembly was decontaminated using the Assembly Screen for Cobionts and Contaminants ( ASCC) pipeline. TreeVal was used to generate the flat files and maps for use in curation. Manual curation was conducted primarily in PretextView and HiGlass ( Kerpedjiev *et al.*, 2018). Scaffolds were visually inspected and corrected as described by Howe *et al*. (2021).

Manual corrections included 21 breaks and 168 joins. This reduced the scaffold count by 13.6%, increased the scaffold N50 by 7.8%, and reduced the total assembly length by 0.6%. The curation process is described at sanger-tol/curation-resources . PretextSnapshot was used to generate a Hi-C contact map of the final assembly.

### Assembly quality assessment

The MerquryFK tool ( Rhie *et al.*, 2020) was run in a Singularity container ( Kurtzer *et al.*, 2017) to evaluate *k*-mer completeness and assembly quality for both haplotypes using the *k*-mer database ( *k* = 31) computed prior to genome assembly. The analysis outputs included assembly QV scores and completeness statistics.

The genome was analysed using the BlobToolKit pipeline, a Nextflow implementation of the earlier Snakemake version ( Challis *et al.*, 2020). The pipeline aligns PacBio reads using minimap2 ( Li, 2018) and SAMtools ( Danecek *et al.*, 2021) to generate coverage tracks. It runs BUSCO ( Manni *et al.*, 2021) using lineages identified from the NCBI Taxonomy ( Schoch *et al.*, 2020). For the three domain-level lineages, BUSCO genes are aligned to the UniProt Reference Proteomes database ( Bateman *et al.*, 2023) using DIAMOND blastp ( Buchfink *et al.*, 2021). The genome is divided into chunks based on the density of BUSCO genes from the closest taxonomic lineage, and each chunk is aligned to the UniProt Reference Proteomes database with DIAMOND blastx. Sequences without hits are chunked using seqtk and aligned to the NT database with blastn ( Altschul *et al.*, 1990). The BlobToolKit suite consolidates all outputs into a blobdir for visualisation. The BlobToolKit pipeline was developed using nf-core tooling ( Ewels *et al.*, 2020) and MultiQC ( Ewels *et al.*, 2016), with containerisation through Docker ( Merkel, 2014) and Singularity ( Kurtzer *et al.*, 2017).

## Genome sequence report

### Sequence data

PacBio sequencing of the *Tadarida teniotis* specimen generated 101.96 Gb (gigabases) from 8.92 million reads, which were used to assemble the genome. GenomeScope2.0 analysis estimated the haploid genome size at 2 411.12 Mb, with a heterozygosity of 0.54% and repeat content of 22.07% ( Figure 2). These estimates guided expectations for the assembly. Based on the estimated genome size, the sequencing data provided approximately 41× coverage. Hi-C sequencing produced 401.63 Gb from 1 329.91 million reads, which were used to scaffold the assembly. RNA sequencing data were also generated and are available in public sequence repositories. Table 1 summarises the specimen and sequencing details.

**Figure 2.**
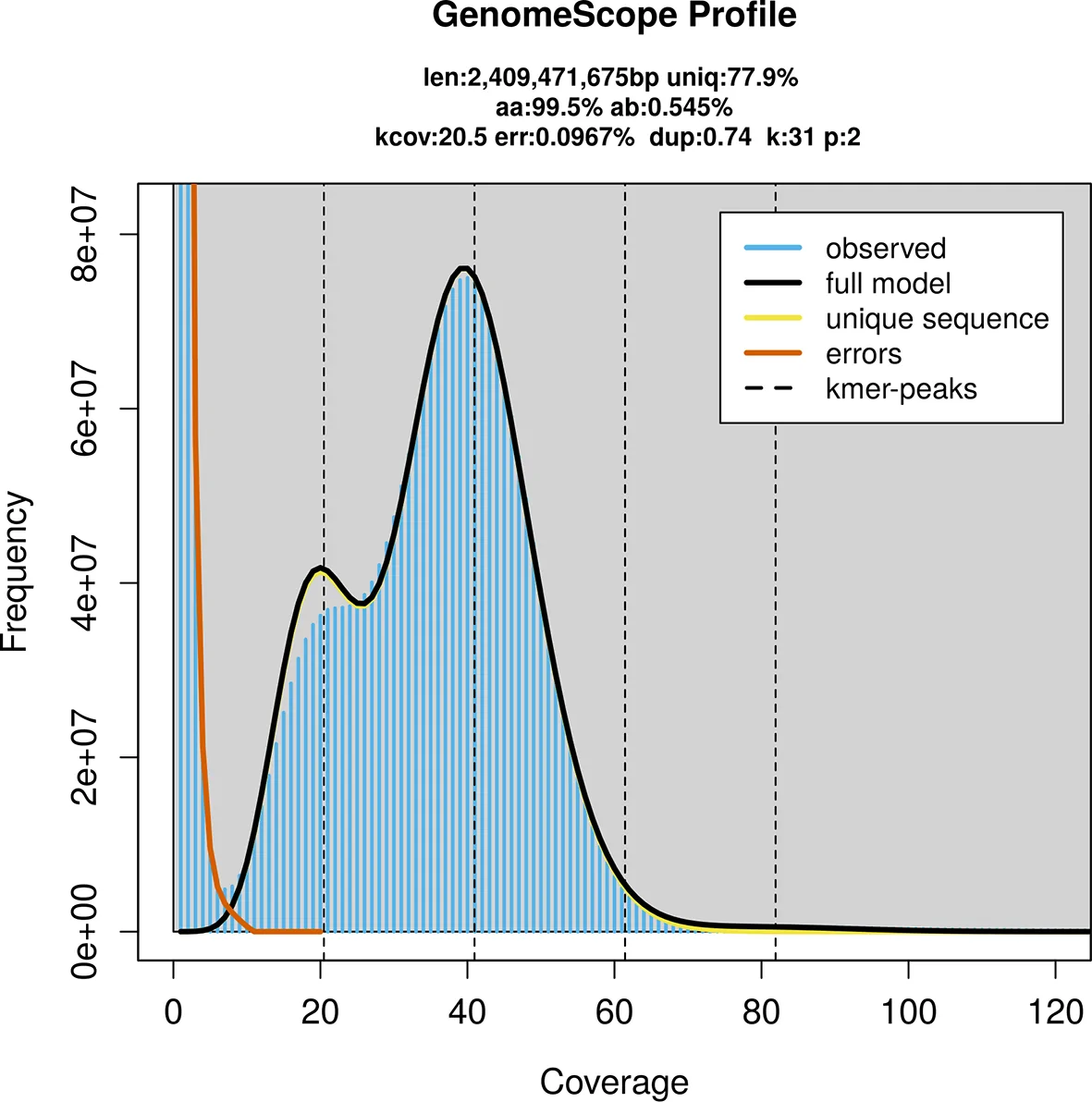
Frequency distribution of *k*-mers generated using GenomeScope2. The plot shows observed and modelled *k*-mer spectra, providing estimates of genome size, heterozygosity, and repeat content based on unassembled sequencing reads.

**Table 1.** Specimen and sequencing data for *Tadarida teniotis* (BioProject PRJEB97030).

Platform	PacBio HiFi	Hi-C	RNA-seq
**ToLID**	mTadTen1	mTadTen1	mTadTen1
**Specimen ID**	SAN27000001	SAN27000001	SAN27000001
**BioSample (source individual)**	SAMEA115534555	SAMEA115534555	SAMEA115534555
**BioSample (tissue)**	SAMEA115534556	SAMEA115534558	SAMEA115534559
**Tissue**	heart	muscle	spleen
**Instrument**	Revio	Illumina NovaSeq X	Illumina NovaSeq X
**Run accessions**	ERR15532267; ERR15532268	ERR15551570	ERR15551569
**Read count total**	8.92 million reads	1 329.91 million read pairs	34.75 million read pairs
**Base count total**	101.96 Gb	401.63 Gb	10.49 Gb

### Assembly statistics

The genome was assembled into two haplotypes using Hi-C phasing. Haplotype 1 was curated to chromosome level, while haplotype 2 was assembled to scaffold level. The final assembly has a total length of 2 587.88 Mb in 640 scaffolds, with 174 gaps, and a scaffold N50 of 115.36 Mb ( Table 2).

**Table 2.** Genome assembly statistics for *Tadarida teniotis.*

Genome assembly	Haplotype 1	Haplotype 2
**Assembly name**	mTadTen1.hap1.1	mTadTen1.hap2.1
**Assembly accession**	GCA_976988885.1	GCA_976988855.1
**Assembly level**	chromosome	chromosome
**Span (Mb)**	2 587.88	2 416.25
**Number of chromosomes**	24	24
**Number of contigs**	814	491
**Contig N50**	53.14 Mb	51.23 Mb
**Number of scaffolds**	640	370
**Scaffold N50**	115.36 Mb	113.62 Mb
**Longest scaffold length (Mb)**	262.7	258.07
**Sex chromosomes**	X	X
**Organelles**	Mitochondrial genome: 16.88 kb	-

Most of the haplotype 1 assembly sequence (94.1%) was assigned to 24 chromosomal-level scaffolds, representing 23 autosomes and the X sex chromosome. These chromosome-level scaffolds, confirmed by Hi-C data, are named according to size ( Figure 3; Table 3). X chromosomes were assigned based on synteny with the genome of *Tadarida brasiliensis* (GCA_030848825.1). During curation we noted that a large number of centromeric repeats are in the unlocalised sequences.

**Figure 3.**
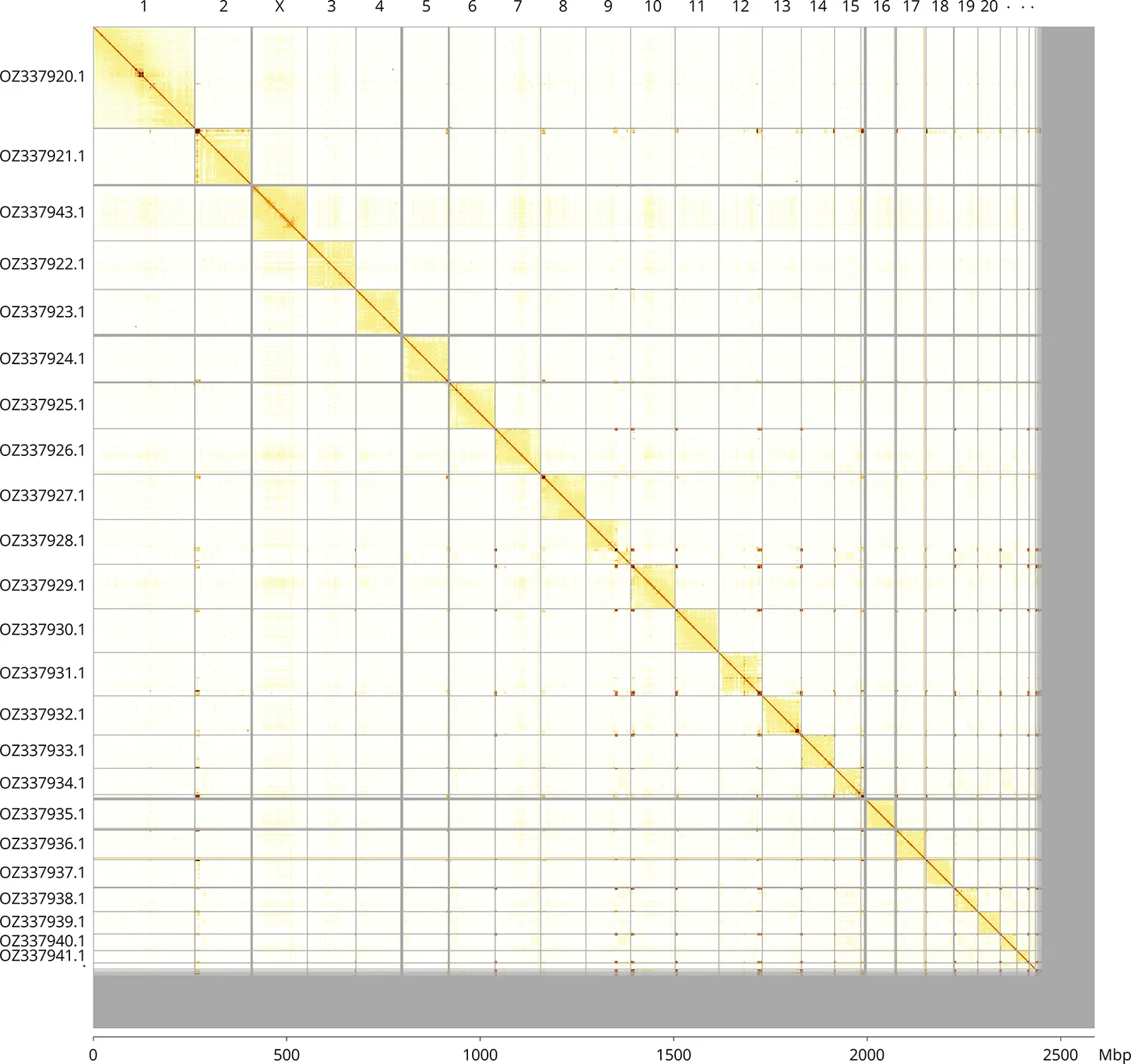
Hi-C contact map of the *Tadarida teniotis* genome assembly. Assembled chromosomes are shown in order of size and labelled along the axes, with a megabase scale shown below. The plot was generated using PretextSnapshot.

**Table 3.** Chromosomal pseudomolecules in both haplotypes of the genome assembly of *Tadarida teniotis*, mTadTen1.

Haplotype 1				Haplotype 2			
INSDC accession	Name	Length (Mb)	GC%	INSDC accession	Name	Length (Mb)	GC%
OZ337920.1	1	263.45	41	OZ337947.1	1	258.07	41
OZ337921.1	2	148.03	42	OZ337948.1	2	143.52	42
OZ337922.1	3	125.85	43	OZ337949.1	3	122	43
OZ337923.1	4	121.33	40	OZ337950.1	4	115.22	40
OZ337924.1	5	120.37	42	OZ337951.1	5	121.21	42
OZ337925.1	6	117.85	40.50	OZ337952.1	6	119.55	40.50
OZ337926.1	7	117.61	42.50	OZ337953.1	7	116.44	42.50
OZ337927.1	8	116.82	40	OZ337954.1	8	113.62	40
OZ337928.1	9	116.02	43.50	OZ337955.1	9	110.55	43.50
OZ337929.1	10	114.78	41	OZ337956.1	10	110.58	40.50
OZ337930.1	11	113.09	40.50	OZ337957.1	11	106	40.50
OZ337931.1	12	112.29	43	OZ337958.1	12	103.89	43
OZ337932.1	13	100.78	43	OZ337959.1	13	95.44	43
OZ337933.1	14	85.96	42.50	OZ337960.1	14	84.05	42.50
OZ337934.1	15	81.90	46	OZ337961.1	15	67.87	46
OZ337935.1	16	78.24	41	OZ337962.1	16	78.53	41
OZ337936.1	17	75.73	43.50	OZ337963.1	17	73.99	43.50
OZ337937.1	18	73.37	43.50	OZ337964.1	18	72.01	43.50
OZ337938.1	19	61.26	46	OZ337965.1	19	59.79	46
OZ337939.1	20	57.54	44.50	OZ337966.1	20	56.30	44.50
OZ337940.1	21	43.05	46.50	OZ337967.1	21	40.66	46.50
OZ337941.1	22	31.54	44	OZ337968.1	22	26.45	44.50
OZ337942.1	23	16.42	47	OZ337969.1	23	15.40	47
OZ337943.1	X	141.89	39.50	OZ337970.1	X	141.15	39.50

The mitochondrial genome was also assembled (length 16.88 kb, OZ337944.1). This sequence is included as a contig in the multifasta file of the genome submission and as a standalone record.

### Assembly quality metrics

For haplotype 1, the estimated QV is 70.9, and for haplotype 2, 71.3. When the two haplotypes are combined, the assembly achieves an estimated QV of 71.1. The *k*-mer ompleteness is 89.75% for haplotype 1, 89.59% for haplotype 2, and 99.57% for the combined haplotypes ( Figure 4).

**Figure 4.**
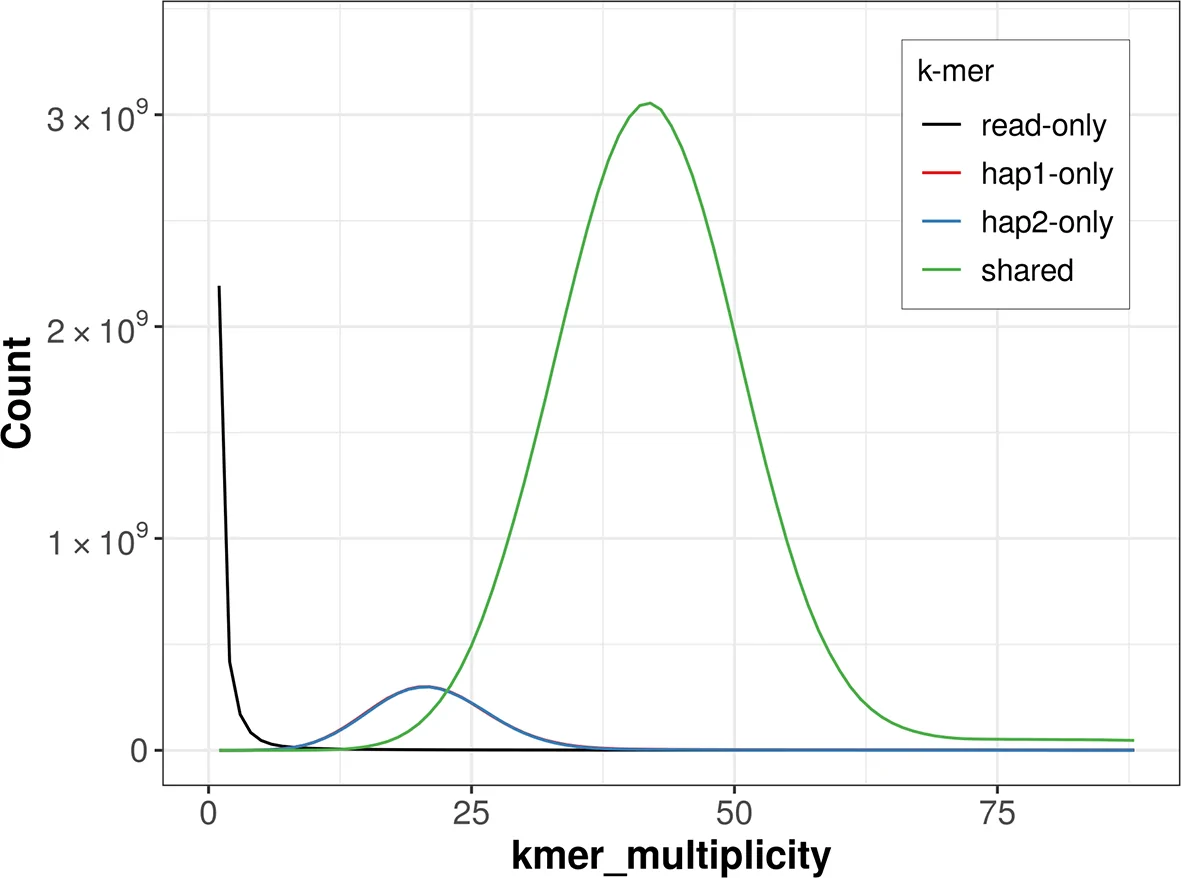
Evaluation of *k*-mer completeness using MerquryFK. This plot illustrates the recovery of *k*-mers from the original read data in the final assemblies. The horizontal axis represents *k*-mer multiplicity, and the vertical axis shows the number of *k*-mers. The black curve represents *k*-mers that appear in the reads but are not assembled. The green curve corresponds to *k*-mers shared by both haplotypes, and the red and blue curves show *k*-mers found only in one of the haplotypes.

BUSCO analysis using the laurasiatheria_odb10 reference set ( *n* = 12 234) identified 99.4% of the expected gene set (single = 97.6%, duplicated = 1.7%) in haplotype 1. For haplotype 2, BUSCO v.6.0.0 analysis identified 99.3% of the expected gene set (single = 97.8%, duplicated = 1.5%). The snail plot in Figure 5 summarises the scaffold length distribution and other assembly statistics for haplotype 1. The blob plot in Figure 6 shows the distribution of scaffolds by GC proportion and coverage for haplotype 1.

**Figure 5.**
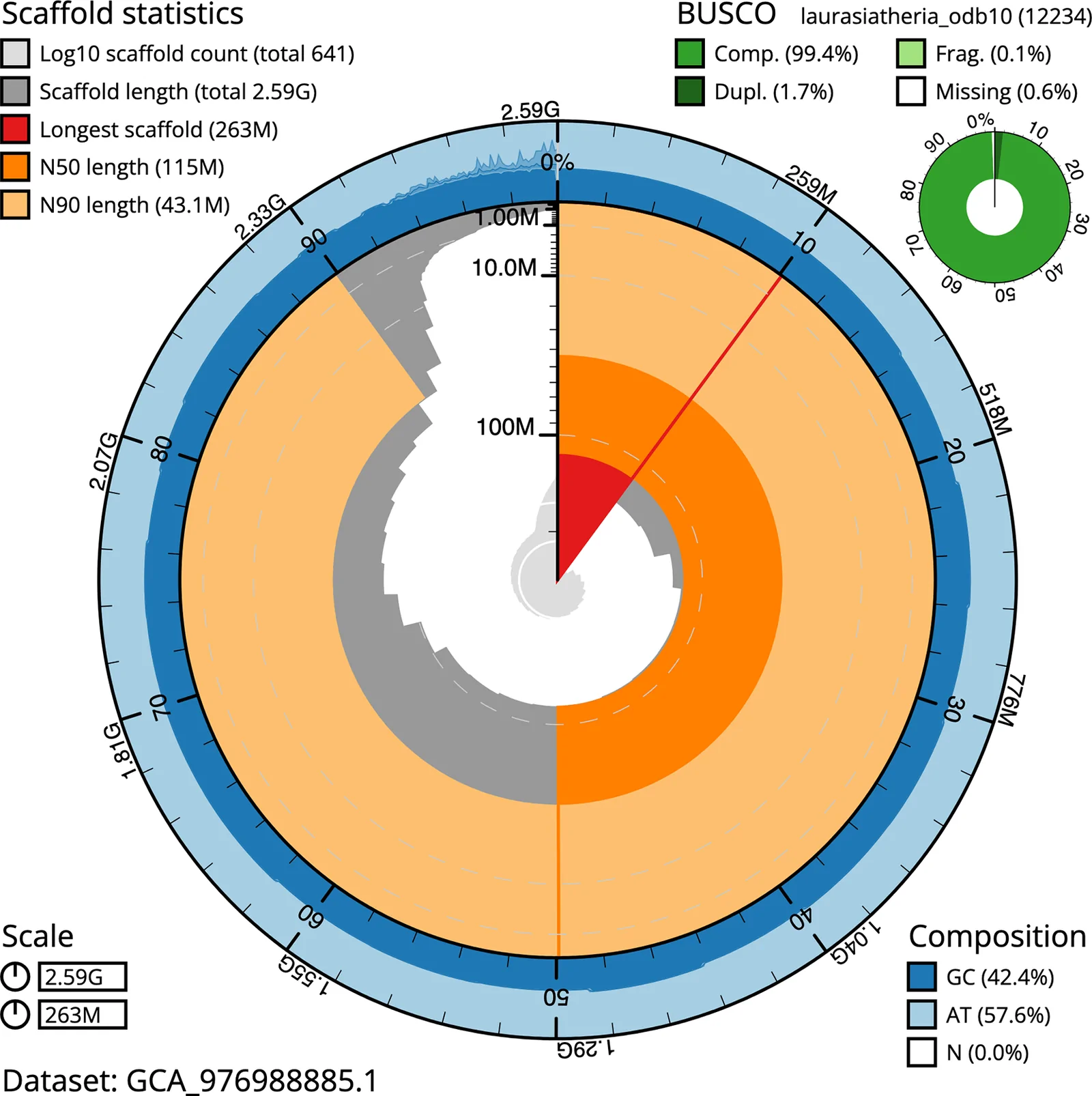
Assembly metrics for mTadTen1.hap1.1. The BlobToolKit snail plot provides an overview of assembly metrics and BUSCO gene completeness. The circumference represents the length of the whole genome sequence, and the main plot is divided into 1 000 bins around the circumference. The outermost blue tracks display the distribution of GC, AT, and N percentages across the bins. Scaffolds are arranged clockwise from longest to shortest and are depicted in dark grey. The longest scaffold is indicated by the red arc, and the deeper orange and pale orange arcs represent the N50 and N90 lengths. A light grey spiral at the centre shows the cumulative scaffold count on a logarithmic scale. A summary of complete, fragmented, duplicated, and missing BUSCO genes in the set is presented at the top right. An interactive version of this figure can be accessed on the BlobToolKit viewer.

**Figure 6.**
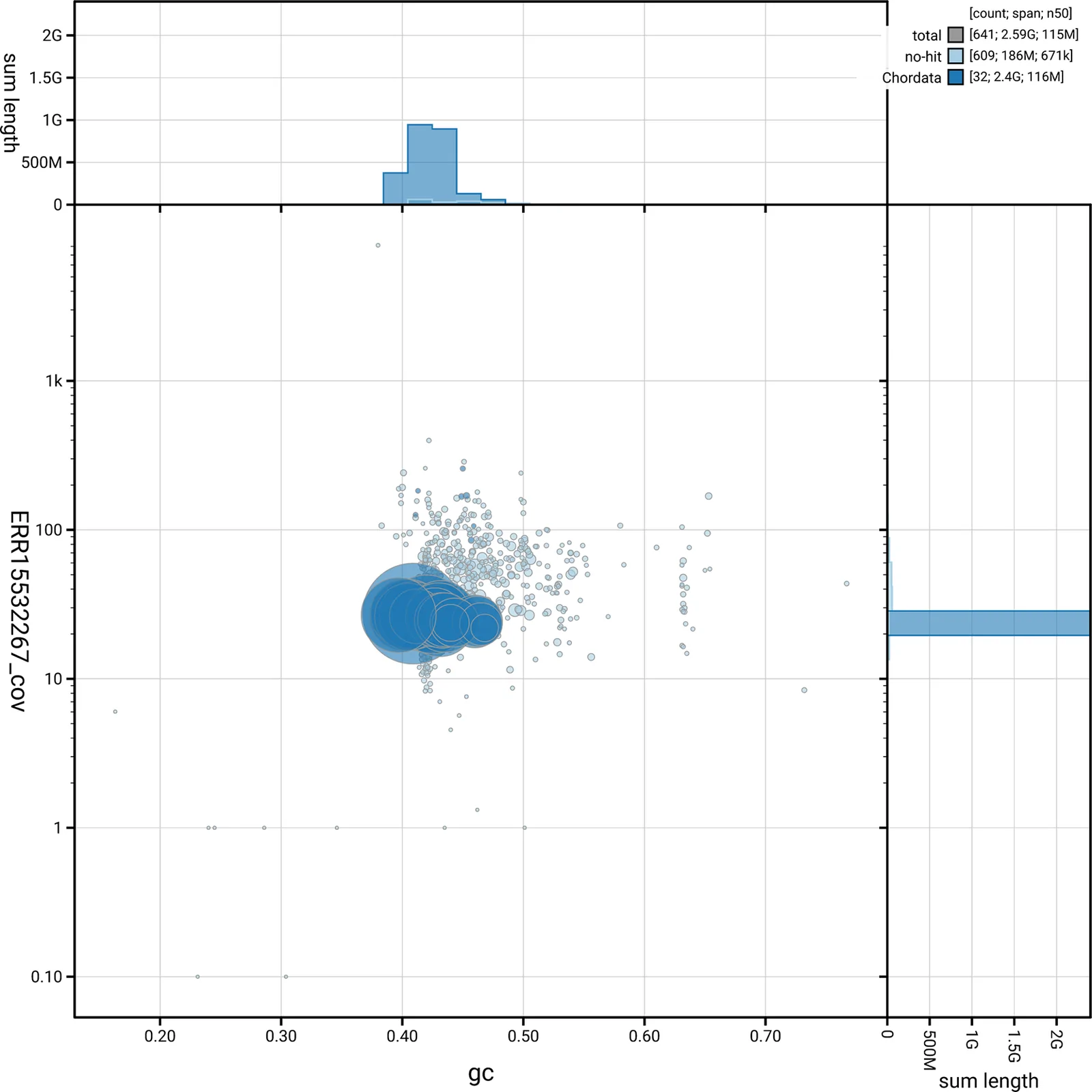
BlobToolKit blob plot for mTadTen1.hap1.1. The plot shows base coverage (vertical axis) and GC content (horizontal axis). The circles represent scaffolds, with the size proportional to scaffold length and the colour representing phylum membership. The histograms along the axes display the total length of sequences distributed across different levels of coverage and GC content. An interactive version of this figure is available on the BlobToolKit viewer.

Table 4 lists the assembly metric benchmarks adapted from Rhie *et al.* (2021) and the Earth BioGenome Project Report on Assembly Standards. The EBP metric, calculated for the haplotype 1, is **7.C.Q70**, meeting the recommended 6.C.Q40 reference standard.

**Table 4.** Earth Biogenome Project summary metrics for the *Tadarida teniotis* assembly.

Measure	Value	Benchmark
EBP summary (haplotype 1)	7.C.Q70	6.C.Q40
Contig N50 length	53.14 Mb	≥ 1 Mb
Scaffold N50 length	115.36 Mb	= chromosome N50
Consensus quality (QV)	Haplotype 1: 70.9; haplotype 2: 71.3; combined: 71.1	≥ 40
*k*-mer completeness	Haplotype 1: 89.75%; Haplotype 2: 89.59%; combined: 99.57%	≥ 95%
BUSCO	Haplotype 1: C:99.4% [S:97.6%, D:1.7%], F:0.1%, M:0.6%, n:12 234; Haplotype 2: C:99.3% [S:97.8%, D:1.5%], F:0.1%, M:0.6%, n:12 234	S > 90%; D < 5%
Percentage of assembly assigned to chromosomes	94.10%	≥ 90%

*Note:* The EBP summary uses log10(Contig N50); chromosome-level (C) or log10(Scaffold N50); Q (Merqury QV). BUSCO: C=complete; S=single-copy; D=duplicated; F=fragmented; M=missing; n=orthologues.

**Table 5.** Software versions and sources used for *Tadarida teniotis.*

Software	Version	Source
BLAST	2.15.0+; 2.16.0+	ftp://ftp.ncbi.nlm.nih.gov/blast/executables/blast+/
BlobToolKit	0.8.0; 4.4.6	https://github.com/blobtoolkit/blobtoolkit
BUSCO	6.0.0	https://gitlab.com/ezlab/busco
bwa-mem2	2.2.1	https://github.com/bwa-mem2/bwa-mem2
DIAMOND	2.1.8	https://github.com/bbuchfink/diamond
fasta_windows	0.2.4	https://github.com/tolkit/fasta_windows
FastK	1.1	https://github.com/thegenemyers/FASTK
GenomeScope2.0	2.0.1	https://github.com/tbenavi1/genomescope2.0
Gfastats	1.3.6	https://github.com/vgl-hub/gfastats
Hifiasm	0.25.0-r726	https://github.com/chhylp123/hifiasm
HiGlass	1.13.4	https://github.com/higlass/higlass
MerquryFK	1.1.0-c1	https://github.com/thegenemyers/MERQURY.FK
Minimap2	2.24-r1122; 2.28-r1209	https://github.com/lh3/minimap2
MitoHiFi	3.2.2	https://github.com/marcelauliano/MitoHiFi
MultiQC	1.14; 1.17 and 1.18	https://github.com/MultiQC/MultiQC
Nextflow	24.10.4; 25.04.6; 25.04.6.5954	https://github.com/nextflow-io/nextflow
PretextSnapshot	0.0.4	https://github.com/sanger-tol/PretextSnapshot
PretextView	1.0.3	https://github.com/sanger-tol/PretextView
samtools	1.17; 1.2; 1.21	https://github.com/samtools/samtools
sanger-tol/ascc	0.1.0	https://github.com/sanger-tol/ascc
sanger-tol/blobtoolkit	v0.9.0	https://github.com/sanger-tol/blobtoolkit
sanger-tol/curationpretext	1.4.2	https://github.com/sanger-tol/curationpretext
Seqtk	1.4-r122	https://github.com/lh3/seqtk
Singularity	3.9.0	https://github.com/sylabs/singularity
TreeVal	v1.4.0	https://github.com/sanger-tol/treeval
YaHS	1.2.2	https://github.com/c-zhou/yahs

## Author information

Contributors are listed at the following links:

• Members of the Wellcome Sanger Institute Tree of Life Management, Samples and Laboratory team
• Members of Wellcome Sanger Institute Scientific Operations – Sequencing Operations
• Members of the Wellcome Sanger Institute Tree of Life Core Informatics team
• Members of the Tree of Life Core Informatics collective
• Members of the Darwin Tree of Life Consortium

## Wellcome Sanger Institute – Legal and Governance

The materials that have contributed to this genome note have been supplied by a Darwin Tree of Life Partner. The submission of materials by a Darwin Tree of Life Partner is subject to the **‘Darwin Tree of Life Project Sampling Code of Practice’**, which can be found in full on the Darwin Tree of Life website. By agreeing with and signing up to the Sampling Code of Practice, the Darwin Tree of Life Partner agrees they will meet the legal and ethical requirements and standards set out within this document in respect of all samples acquired for, and supplied to, the Darwin Tree of Life Project. Further, the Wellcome Sanger Institute employs a process whereby due diligence is carried out proportionate to the nature of the materials themselves, and the circumstances under which they have been/are to be collected and provided for use. The purpose of this is to address and mitigate any potential legal and/or ethical implications of receipt and use of the materials as part of the research project, and to ensure that in doing so we align with best practice wherever possible. The overarching areas of consideration are:

• Ethical review of provenance and sourcing of the material
• Legality of collection, transfer and use (national and international)

Each transfer of samples is further undertaken according to a Research Collaboration Agreement or Material Transfer Agreement entered into by the Darwin Tree of Life Partner, Genome Research Limited (operating as the Wellcome Sanger Institute), and in some circumstances, other Darwin Tree of Life collaborators.

## Data Availability

European Nucleotide Archive: Tadarida teniotis (European free-tailed bat). Accession nk,number PRJEB97030; https://identifiers.org/ena.embl/PRJEB97030. The genome sequence is released openly for reuse. The *Tadarida teniotis* genome sequencing initiative is part of the Sanger Institute Tree of Life Programme (PRJEB43745), the Vertebrate Genomes Project (PRJNA489243) and the Bat1K Project (PRJNA489245). All raw sequence data and the assembly have been deposited in INSDC databases. The genome will be annotated using available RNA-Seq data and presented through the Ensembl pipeline at the European Bioinformatics Institute. Raw data and assembly accession identifiers are reported in Tables 1 and 2. Production code used in genome assembly at the WSI Tree of Life is available at https://github.com/sanger-tol . Table 5 lists software versions used in this study.
