# Supplementary material for: The genome sequence of the European Free-tailed Bat, Tadarida teniotis (Rafinesque, 1814) (Chiroptera: Molossidae)
Source: Wellcome Open Res. 2026 Jul 21;11:454. [Version 1] doi: 10.12688/wellcomeopenres.27076.1 (PMC13675633; doi:10.12688/wellcomeopenres.27076.1)
Supplement: Peer review history

# Reviewer report: Reviewer response for version 1

**DOI:** 10.21956/wellcomeopenres.29802.r171739

**Published:** 2026-10-16

**Authors:** Xiuguang Mao

**Affiliations:** 1 East China Normal University, Shanghai, China

This manuscript presents a high-quality, chromosome-scale genome assembly for *Tadarida teniotis*, generated in accordance with the standards of the Bat1K sequencing project. I have one minor comment: " *Tadarida teniotis*" should be italicized in the keywords section. I have no further comments on the current version of the manuscript. I thank the authors for their contribution to bat research.

Are sufficient details of methods and materials provided to allow replication by others?

Yes

Is the rationale for creating the dataset(s) clearly described?

Yes

Are the datasets clearly presented in a useable and accessible format?

Yes

Are the protocols appropriate and is the work technically sound?

Yes

Reviewer Expertise:

Comparative genomics, genome assembly, evolutionary biology, speciation, bats

I confirm that I have read this submission and believe that I have an appropriate level of expertise to confirm that it is of an acceptable scientific standard.

# Reviewer report: Reviewer response for version 1

**DOI:** 10.21956/wellcomeopenres.29802.r163471

**Published:** 2026-08-18

**Authors:** Heliana Dundarova

**Affiliations:** 1 Bulgarian Academy of Sciences, Sofia, Bulgaria

This manuscript presents a significant, chromosome-scale, haplotype-resolved genome assembly of the European free-tailed bat, *Tadarida teniotis*, generated using PacBio HiFi sequencing, Hi-C scaffolding, and complementary RNA-seq data. The study provides a valuable genomic resource for comparative genomics, evolutionary biology, and future functional studies of bats, and the assembly is supported by strong quality metrics, including high consensus accuracy (QV ~71), excellent BUSCO completeness (~99.3–99.4%), and 94.1% of haplotype 1 assigned to chromosome-level scaffolds. The overall experimental and computational strategy is appropriate, and the public availability of the raw sequencing data and assemblies substantially enhances the value and reproducibility of the work.

Are sufficient details of methods and materials provided to allow replication by others?

Yes

Is the rationale for creating the dataset(s) clearly described?

Yes

Are the datasets clearly presented in a useable and accessible format?

Yes

Are the protocols appropriate and is the work technically sound?

Yes

Reviewer Expertise:

Chiroptera, phylogeny, ecology, conservation

I confirm that I have read this submission and believe that I have an appropriate level of expertise to confirm that it is of an acceptable scientific standard.
